# Management of overlapping immune-related myocarditis, myositis, and myasthenia in a young patient with advanced NSCLC: a case report

**DOI:** 10.3389/fonc.2024.1431971

**Published:** 2024-10-11

**Authors:** Monica Mariniello, Giulia Arrivi, Laura Tufano, Antonio Lauletta, Mirella Moro, Giacomo Tini, Matteo Garibaldi, Raffaele Giusti, Federica Mazzuca

**Affiliations:** ^1^ Medical Oncology Unit, Sant’Andrea Hospital of Rome, Rome, Italy; ^2^ Department of Clinical and Molecular Medicine, Oncology Unit, Sant’ Andrea University Hospital, Sapienza University of Rome, Rome, Italy; ^3^ PhD School in Translational Medicine and Oncology, Department of Medical and Surgical Sciences and Translational Medicine, Faculty of Medicine and Psycology, Sapienza University of Rome, Rome, Italy; ^4^ Department of Neurology, Mental Health and Sensory Organs (NESMOS), Sapienza University of Rome, Rome, Italy; ^5^ Cardiology, Department of Clinical and Molecular Medicine, Sapienza University of Rome, Rome, Italy; ^6^ Neuromuscular Disease Centre, Sant’Andrea Hospital, Rome, Italy

**Keywords:** immune check inhibitor (ICI), myositis, myocarditis, myasthenia gravis, corticosteriods, immunoglobulin, lung cancer, irAEs (immune-related adverse events)

## Abstract

Immunotherapy is increasingly used in advanced non-small-cell lung cancer (NSCLC), offering a significant anti-tumor response, as well as causing rising immune-related adverse effects. The incidence of immune checkpoint inhibitor-induced myocarditis–myositis–myasthenia gravis is increasing and particularly concerning due to its high mortality rate. Prompt recognition, diagnosis, and management are crucial. A 40-year-old patient, diagnosed with stage IV non-oncogene addicted lung adenocarcinoma, with nivolumab–ipilimumab–chemotherapy as first-line treatment, developed a rare myocarditis–myositis–myasthenia gravis overlap syndrome. Following the treatment, the patient presented with flu-like symptoms and chest pain and subsequently transferred to the cardiac intensive care unit. The physical examination revealed a visual acuity deficit, diplopia, ophthalmoparesis, ptosis, mydriasis, dysphagia, dyspnea, headache, nausea, dry mouth, asthenia, myalgia, and muscle weakness. Imaging and laboratory tests confirmed the triad, showing an elevation of hs-cTnI and CK and positive results for anti-SAE1 and anti-PL-7 Abs. ECG revealed ST segment elevation and RBBB. The echo showed hyperechogenicity of the inferolateral wall, pericardial detachment, and thickening. The cardiac MRI demonstrated hypokinesia, edema, subepicardial LGE, and pericardial effusion. Muscle biopsy revealed muscle fiber necrosis and regeneration with B and T lymphocytic endomysial inflammatory infiltrate and expression of MHC-I. Treatment with oral prednisone, pyridostigmine, and IV Igs was started due to poor clinical response followed by methylprednisolone. Despite stopping immunotherapy, the patient continued to benefit from it, as highlighted on subsequent re-evaluation CT scans by partial disease response, and as the patient was in complete remission, we decided to resume chemotherapy by omitting immunotherapy. At the radiological control following the four cycles of double CHT and during CHT maintenance, there was a further reduction of the disease. This report aims to raise awareness among physicians about these serious side effects. A multidisciplinary approach led to clinical improvement and early intervention, optimizing patient outcomes.

## Introduction

The number of cases of immune checkpoint inhibitor (ICI) overlap syndrome involving myasthenia gravis, myositis, and myocarditis is increasing in the published literature. ICI-associated myocarditis is particularly concerning due to its high mortality rate of nearly 50% and significant cardiovascular complications in up to 46% of cancer patients. The incidence of immune-related myocarditis is approximately 1%, and among the cases, 25% and 11% exhibit concurrent myositis and myasthenia gravis, respectively. Physicians should be familiar with this triad because it requires the prompt discontinuation of ICIs and the timely administration of high-dose glucocorticoids, which represents the mainstay of treatment. In advanced non-small-cell lung cancer (NSCLC), non-oncogene addicted, first-line strategies have shifted to immunotherapy-based treatments, driven by improvements in overall survival compared to standard platinum-based chemotherapy. However, the increasing use of ICIs is accompanied by a rise in toxicity, known as immune-related adverse effects (irAEs), resulting from the disruption of immune checkpoint signaling with ICIs. Any organ system may be affected by irAEs, which may differ in severity and onset (6). We focus on the challenges of recognizing, diagnosing, and treating concomitant myocarditis–myositis– myasthenia gravis associated with ICI.

### Insight

Because of the expanding use of immunotherapy into different tumors, a deeper understanding of immune-related toxicities, their impact on outcomes, development risk factors, and appropriate treatment strategies beyond steroids appears to be crucial. Recognizing the limitations of clinical trials, real-world data are increasingly filling knowledge gaps.

## Case presentation

**Figure 1:**
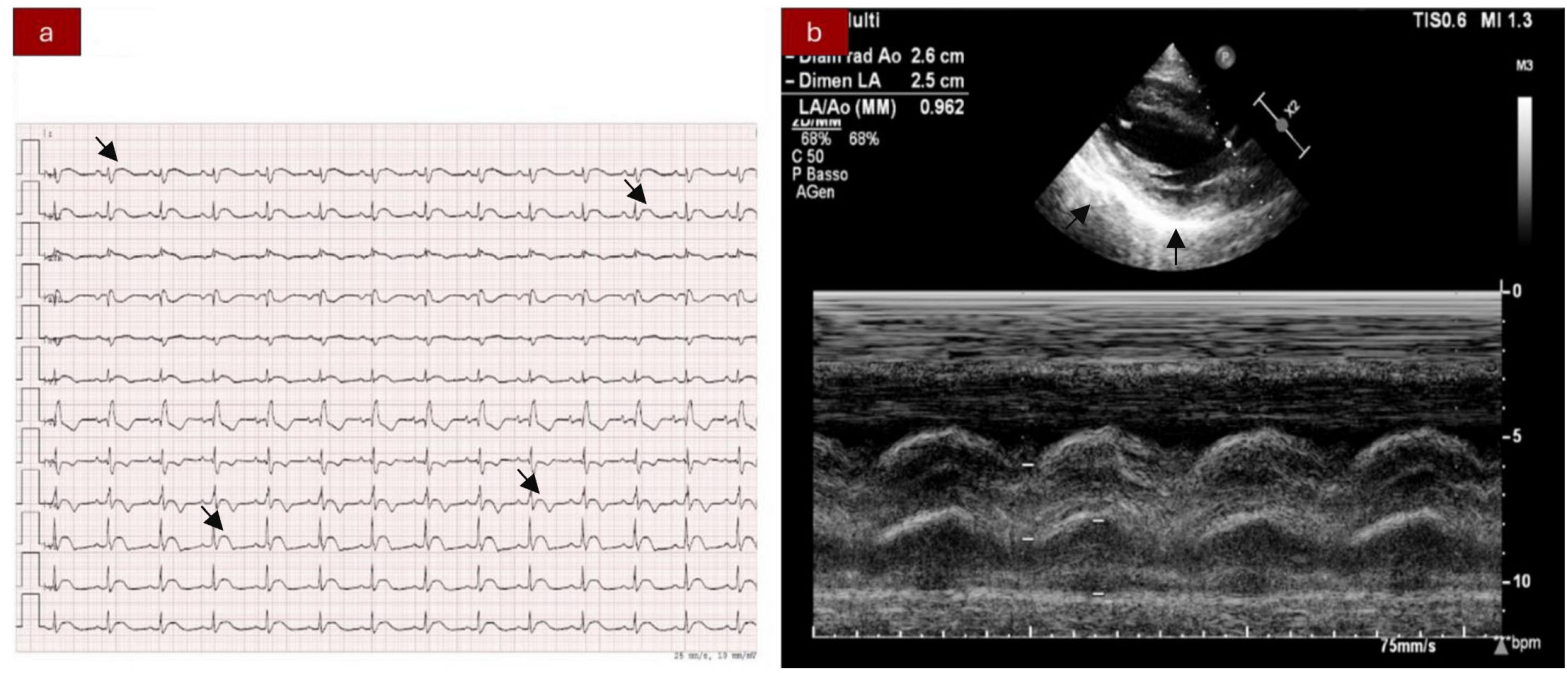
**(A)** Electrocardiogram showing ST segment elevation in inferior-lateral and V3–V6 leads. Right bundle branch block. **(B)** Echocardiography abnormalities: hyperechogenicity of the inferior-lateral wall, mild anterior pericardial detachment, and thickening.

**Figure 2:**
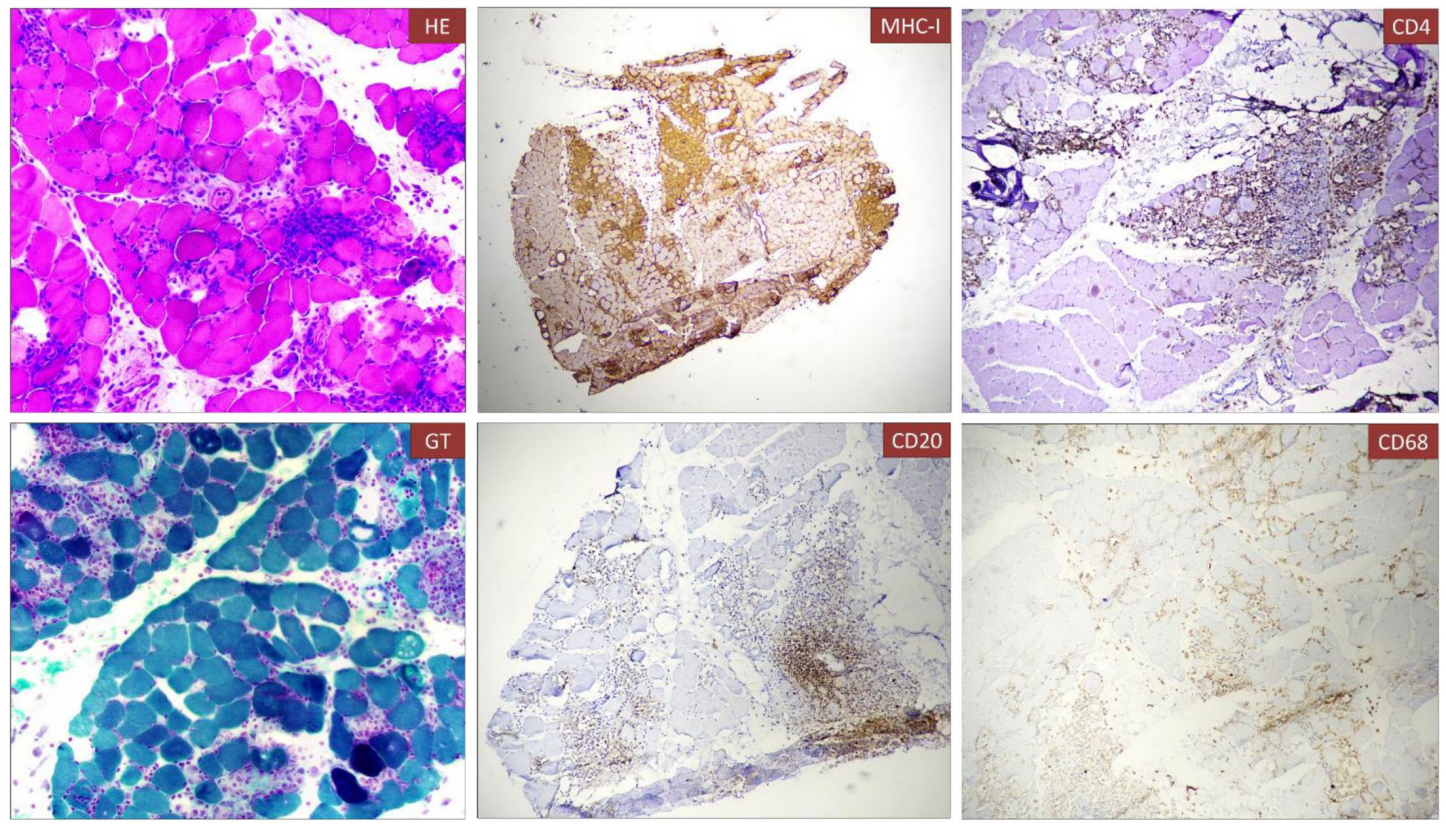
Histological analysis of muscle biopsy. Muscle fiber necrosis and regeneration with marked B and T lymphocyte inflammatory infiltrate (CD4=CD8=CD68), diffuse expression of MHC-I. Immune infiltrate can be observed in the muscle biopsy associated to necrotic fibers [A, Hematoxylin and eosin (H&E)]. The infiltrate was composed partially by T cells (CD4, C) and B-cell lymphocytes (CD20, E).

**Figure 3:**
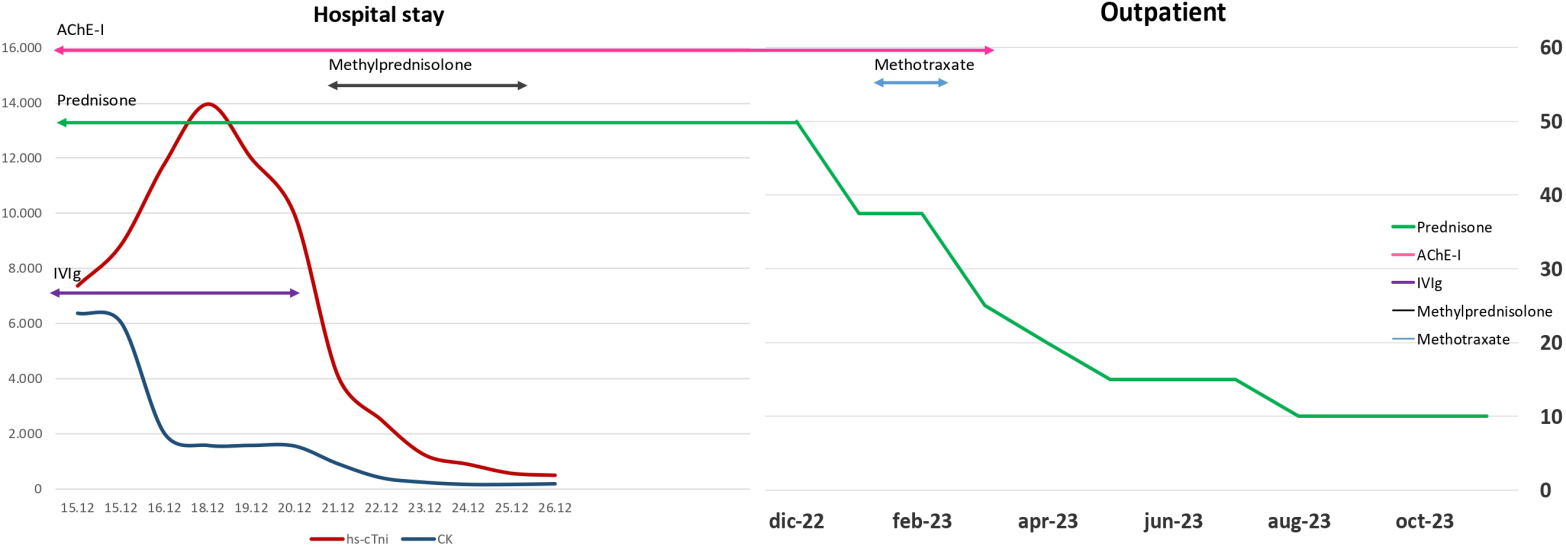
Change in levels of blood indicators of cardiac and muscle damage.

**Figure 4:**
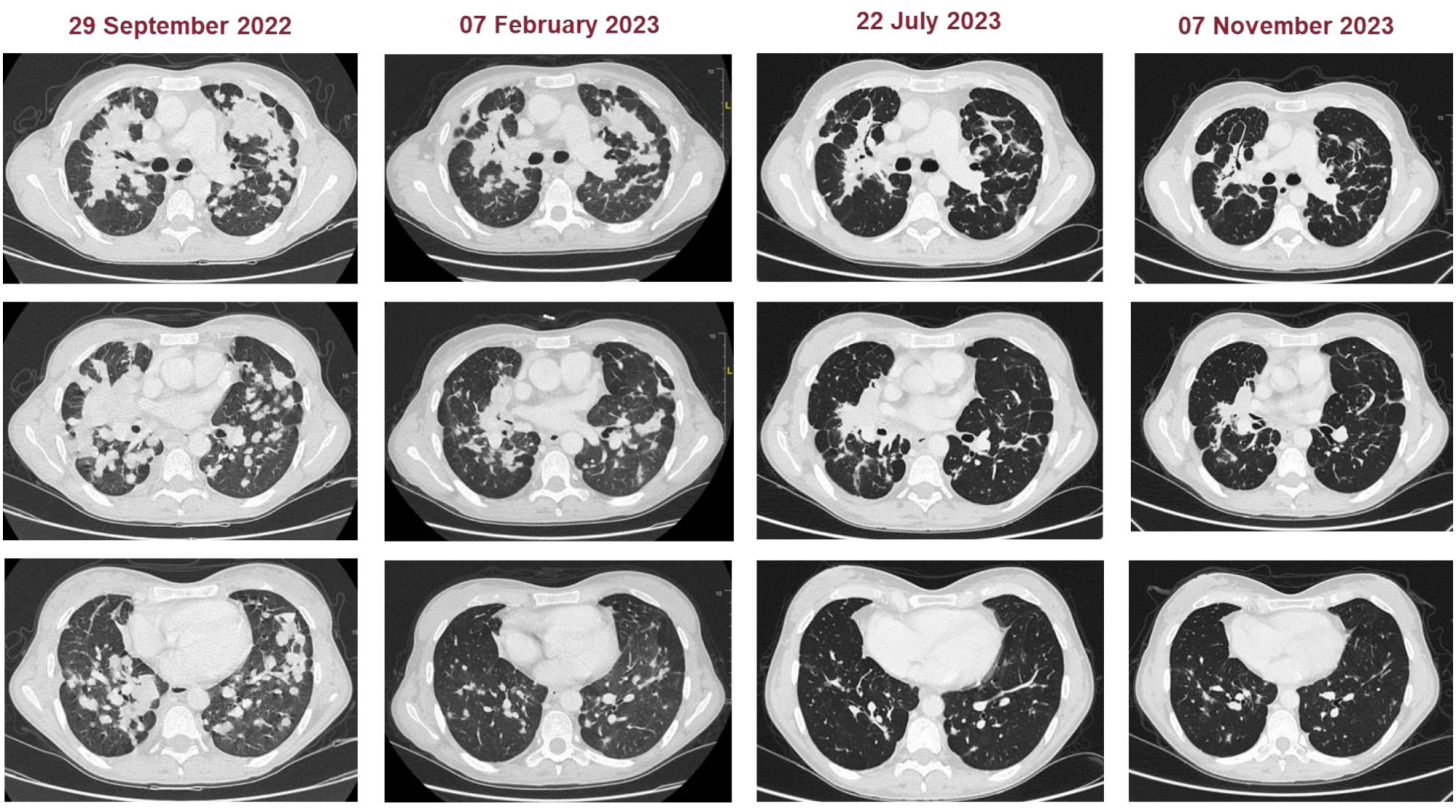
Representative CT images of patients at baseline and during the treatment (subsequently to chemoimmunotherapy, double chemotherapy, and maintenance chemotherapy).

**Figure 5:**
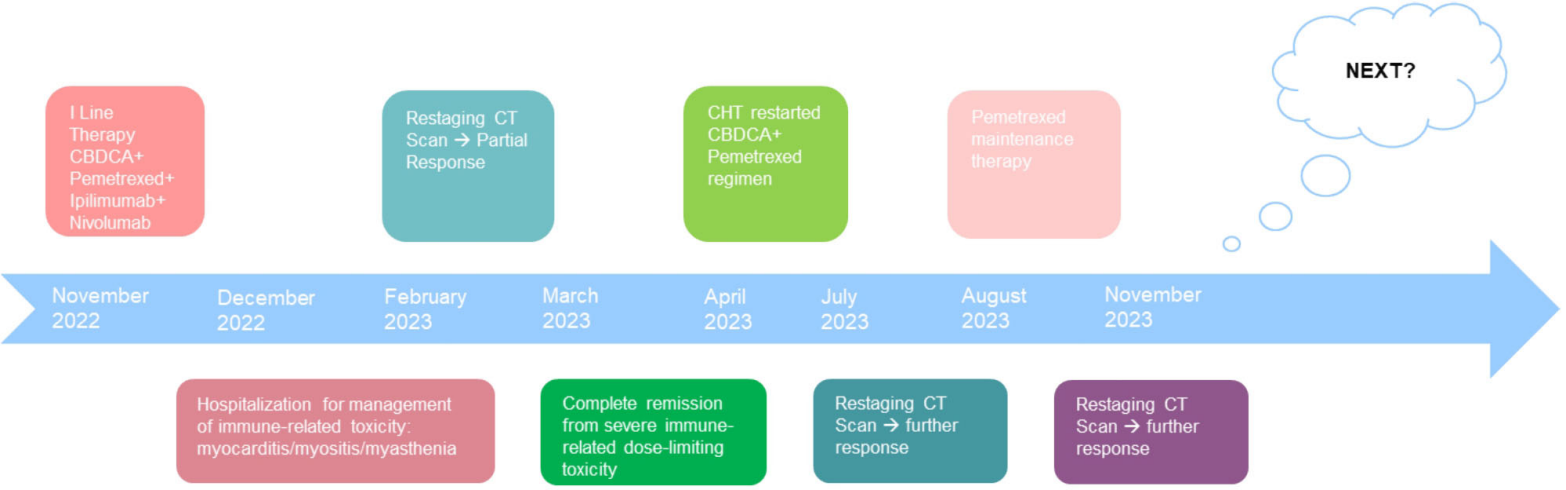
Patient timeline chart along with key dates for investigations and oncological treatments. Although our patient stopped treatment due to severe immuno-related toxicity, the patient had a partial disease response highlighted at the CT revaluation in February 2023. Five months later, however, the patient continued to benefit from treatment with a single administration of four drugs. In March, the patient was in complete remission from severe dose-limiting toxicity. In view of the high risk of disease progression, it was decided to resume chemotherapy treatment by omitting immunotherapy. At the end of the four chemotherapy cycles at the radiological control in July and in November, there was a further reduction of the disease.

We present the case of a 40-year-old Caucasian woman, a former smoker of 2.5 pack-year with an Eastern Cooperative Oncology Group Performance Status (ECOG PS) of 0. Her past medical history includes Hashimoto’s thyroiditis managed with levothyroxine. She had no surgical history and no family history of lung cancer. In October 2022, the patient presented to her general practitioner with persistent cough, dyspnea, and right hemithorax pain [Numeric Range Scale (NRS) = 6]. The patient was diagnosed with stage IV lung adenocarcinoma with no driver mutations and programmed death-ligand (PD-L1) expression <1%. Baseline computed tomography (CT) scan revealed an extensive solid tissue with irregular margins of 5.5 cm in the right perihilar area, with occlusion of the bronchial branch for the middle lobe. It also showed numerous solid nodular formations ranging from 6 to 36 mm, lymphadenopathy in the ilo-mediastinal area of approximately 2 cm, and morphological alteration of the XII right rib. Radiological staging according to the TNM staging VIII Edition system was cT4cN2cM1b Stage IVA (1). The patient underwent diagnostic EBUS-TBNA of the lower paratracheal and subcarinal mediastinal lymph nodes. The subject presented at our oncological outpatient department, and considering the absence of driver alterations, the negative value of PD-L1, and the need to start a systemic treatment to counteract oncological disease progression, we chose first-line systemic chemoimmunotherapy, a combination checkpoint blockade with nivolumab 360 mg every 3 weeks plus ipilimumab 1 mg/kg every 6 weeks, along with carboplatin AUC 5 + pemetrexed 500 mg/m^2^ administered every 3 weeks for two cycles (2). Lipegfilgrastim, a G-CSF, was administered as primary prophylaxis of febrile neutropenia. Additionally, palliative radiation therapy was performed for symptomatic bone lesions on the right XII rib, delivering a total dose of 25 Gray in five fractions. However, the subject only received one cycle of the four-drug combination in November 2022, because in December 2022, the patient reported flu-like symptoms and retrosternal chest pain after treatment and was admitted to the oncology department of our center in Sant’Andrea Hospital of “La Sapienza” University of Rome and subsequently transferred to the cardiac intensive care unit for continuous monitoring. The physical examination revealed a visual acuity deficit, diplopia, severe ophthalmoparesis, left eyelid ptosis, mydriasis, rhinolalia, dysphagia, dyspnea, headache, nausea, dry mouth, asthenia, myalgia, neck flexion deficit, and extensor digitorum communis strength deficit. To exclude a secondary brain metastasis, a brain MRI was performed, which had negative results. Electrocardiogram revealed sinus rhythm, normal atrioventricular conduction, ST segment elevation in inferolateral and V3–V6 leads, and right bundle branch block (**Figure 1A**). Echocardiogram showed normal left ventricular ejection fraction, hyperechogenicity of inferolateral wall, mild anterior pericardial detachment, and thickening (**Figure 1B**). Laboratory tests revealed that Troponin I (hs-cTnI) was persistently elevated up to 13,963 U/L, without dynamic changes. The cardiac MRI confirmed the diagnosis of myocarditis demonstrating LVEF within normal values with mild hypokinesia of the lateral wall, edema, subepicardial late gadolinium enhancement at the level of the lateral wall and antero-lateral to the mid-apical segments, and minimal pericardial effusion at the basal level of the left ventricle. Laboratory tests also showed an increase in creatinine kinase (CK) up to 6,388 U/L and positive results for anti-SAE1 and anti-PL-7 antibodies. Open muscle biopsy was performed on the extensor digitorum communis revealing muscle fiber necrosis and regeneration with marked B and T lymphocytic endomysial inflammatory infiltrate (CD4=CD8=CD68) and diffuse expression of MHC-I, consistent with inflammatory myopathy (**Figure 2**). Concerning neuromuscular transmission assessment, the myasthenia-associated antibodies (anti-AChR and anti-MuSK) and repetitive nerve stimulation were negative, but the ice-pack test was clinically positive. The diagnosis of immune-related myocarditis–myasthenia–myositis association was made, and according to the guidelines (9), we started a therapy with oral prednisone at 1 mg/kg, pyridostigmine bromide 60 mg (one tablet for 4 days), and IV immunoglobulins at 0.4 g/kg daily for 4 days. Despite these interventions, poor clinical response and high troponin values persisted. Moreover, frequent ventricular ectopic beats, with bigeminy, were detected at ECG monitoring. Following a multidisciplinary discussion, therapy with methylprednisolone sodium succinate 1 g IV/day for 5 days was initiated, resulting in an accelerated decline in cTnI and CK levels and the disappearance of arrhythmias (**Figure 3**). The patient remained hemodynamically stable with marked improvement of myocarditis and myositis in the early phase and slow improvement of ophthalmoparesis in the following months. After 2 weeks of hospital stay and treatment, our patient showed a significant symptomatic recovery accompanied by a decrease in myocardial and skeletal muscle injury markers. Anti-PD1 and anti-CTLA 4 therapies were permanently discontinued for the manifestations of grade 3–4 adverse events and no other specific anti-tumor therapy was initiated at that time. Owing to persistent asthenia, the case was discussed with a neurologist colleague, leading to the initiation of methotrexate at 7.5 mg weekly. However, after 2 weeks, the treatment was suspended due to hepatic toxicity. In February 2023, restaging imaging following suspension of ipilimumab–nivolumab treatment revealed a partial response of disease. In March 2023, the patient was in remission from severe dose-limiting toxicity associated with previously carried out immunotherapy with regression of diplopia, dysphagia, bilateral ptosis, and asthenia. Blood tests were within normal limits. The dose of prednisone was tapered and pyridostigmine was suspended. Considering the improvement in general conditions, the patient was advised to resume chemotherapy according to the scheme CBDCA AUC5 plus pemetrexed 500 mg/m^2^ every 3 weeks for four cycles. Subsequent restaging imaging following completion of treatment showed further reduction of disease. Thus, pemetrexed maintenance therapy was started and it is still ongoing. The patient underwent the last radiographic examination in November 2023, revealing continued partial disease response (**Figures 4**, **5**).

## Discussion

ICIs are novel therapeutic agents that have demonstrated relevant anti-tumor responses in various cancer types by enhancing the immune response against cancer and blocking the negative downregulation of T cells. As ICIs can lead to severe side effects and are increasingly used in subspecialties besides oncology, with less experience with these drugs, knowledge should be spread (3). The aim of this article is to raise awareness among physicians about the serious side effects of immune checkpoint blocking antibodies. A comprehensive assessment of the risk of irAEs in patients as early as possible will significantly improve the prognosis of our patients, considering that irAEs can lead to severe consequences. IrAEs caused by ICIs should be paid attention to from many aspects, and before their use, we suggest to comprehensively evaluate whether the patients have the risk factors for irAEs (6–8). Furthermore, it must be considered that ICIs are often used in combination with several drugs that may increase all the risks, mostly of cardiovascular disease.

In comparison to patients treated with nivolumab alone, those treated with nivolumab plus ipilimumab have an increased incidence and severity of myocarditis (up to 2%) (4, 5). The myasthenia symptoms of the patient we reported gradually worsened. The patient believed that it was due to the further development of the tumor and did not pay enough attention to it, which increased the difficulty of later treatment. Therefore, we need to improve the cognition of patients and their families regarding the severity of irAEs, make a clear diagnosis as soon as possible, and facilitate further treatment.

Recent guidelines from AIOM and ESMO (9) provide key recommendations for recognizing and managing acute irAEs.

Diagnosing myocarditis can be challenging due to the absence or the non-specificity of cardiovascular symptoms; thus, initial assessment includes cardiac enzymes, ECG, and echocardiogram. Most importantly, a high level of suspicion is required when managing these patients; otherwise, cases may be undiagnosed. Findings that should raise the suspicion of myocarditis include elevated troponin T and CK levels, cardiac conduction alterations, and ventricular size or function abnormalities. Cardiac imaging with MRI or even endomyocardial biopsy might be necessary for diagnosis confirmation. Given the high mortality rate, hospitalization for clinical monitoring and treatment in severe cases and management in a specialist cardiological environment may be useful in the case of suspected myocarditis (2, 12).

The clinical manifestations of myositis are the result of oculomotor and axial muscle involvement, leading to diplopia, ptosis, and drooping head, while bulbar muscle involvement can cause dysarthria, dysphonia, or dysphagia. Blood chemistry may show elevated muscle damage markers (CPK, LDH, and transaminase) and myositis-specific or myositis-related antibodies, although these are frequently negative. In ICI-related cases, frequent involvement of other peripheral nervous system targets (MG) is reported, detectable through examinations like the EMG/ENG, muscle MRI, and biopsy. Severe cases are linked to the extension of the myopathic process to the myocardium and diaphragm with the onset of myocarditis and respiratory failure with high lethality.

Myasthenia gravis during ICI treatment presents as a severe and high-mortality pathology, compared to idiopathic forms. Generally, it occurs *de novo* and two-thirds of patients have positive antibodies to acetylcholine receptor. The typical clinical picture is characterized by muscle weakness with exhaustion and fluctuation; eye involvement (ptosis and diplopia) and bulbar (dysphagia, dysphonia, dysarthria) or generalized forms are recognized (spinal innervation muscle deficiency). The low-frequency repetitive nerve stimulation (3–5 Hz) has a high specificity but a lower sensitivity, showing the typical decremental response; single-fiber electromyography with the jitter study has a higher sensitivity but a lower specificity. High-title anti-AChR antibodies are recognized in approximately 80%–85% of cases not related to ICI; in ICI-related forms, a higher incidence of seronegative forms is reported. Involvement of skeletal muscle and myocardium is significantly frequent. An emerging complication during ICI therapy is the appearance of an immune attack on multiple targets of the peripheral nervous system and muscle, giving rise to “overlap” or “combined” forms (myasthenia + myositis + polyneuropathy + myocarditis). The most common form of association appears to be between MG and myositis, often associated with at least subclinical myocardial involvement (i.e., asymptomatic elevation of hsTnI). These forms appear substantially exclusive of a related ICI etiology and are associated with higher severity and mortality compared to individual syndromes.

Management of clinically significant irAEs begins with discontinuation of ICI treatment and initiation of corticosteroids, typically prednisone at a dose of 1 to 2 mg/kg or equivalent. In non-responsive patients, increasing corticosteroid dose with intravenous methylprednisolone at a daily dose of 1–2 mg/kg or 500–1,000 mg and/or the addition of other immunosuppressive agents (MMF or tocilizumab 8 mg/kg) or using third-line drugs (such as ATG, alemtuzumab, or abatacept) is usually effective in controlling irAEs. Other therapeutic approaches include adalimumab, infliximab, mycophenolate, thymoglobulin, plasmapheresis, and intravenous immunoglobulin (0.4 g/kg/day for 5 days). Acetylcholinesterase inhibitors pyridostigmine bromide 60 mg (one tablet for 4 days) should be considered in the case of myasthenia gravis, and some studies have shown that plasma exchange is more effective.

**Table 1** shows similar cases of ICI-related myocarditis and myositis/myasthenia gravis overlap syndrome reported in the literature, categorized by diagnosis, treatment, time of onset in weeks from the start of therapy, outcome, and best radiological disease response.

**Table 1:** Reported cases of ICI-related myocarditis and myositis/myasthenia gravis overlap syndrome.

Reference	Type of malignancy	ICI	Time to onset (weeks)	In-hospital outcome	Best response to ICI
Xing, 2020 (13)	NSCLC	Sintilimab	3–4	Alive	PD
Valenti-Azcarate, 2020 (7)	NSCLC	Nivolumab + Ipilimumab	4	Dead	PD
Fukasawa, 2017 (14)	NSCLC	Nivolumab	7	Alive	SD
Fazel, 2019 (15)	Melanoma	Nivolumab + Ipilimumab	1	Dead	PD
Yin, 2022 (16)	Cholangiocarcinoma	Sintilimab	1	Alive	SD

Despite the adverse effects, the nivolumab–ipilimumab–chemotherapy combination now represents a first-line standard for treating advanced non-oncogene addicted lung cancer, either squamous or non-squamous histology. The Checkmate 9LA study, published in *Lancet Oncology* in January 2021, showed superior efficacy and activity in naive advanced NSCLC non-oncogene addicted patients treated with nivolumab (anti-PD1) plus ipilimumab (anti-CTLA4) associated with two cycles of platinum doublet chemotherapy histology based on their subsequent maintenance compared to four cycles of standard chemotherapy and subsequent maintenance with pemetrexed in non-squamous histology. The use of a double immunological block and the lower amount of chemotherapy differentiate this scheme from the therapeutic standard. The most common treatment-related adverse events were neutropenia 7% in the experimental arm vs. 9% in the control arm, anemia 6% vs. 14%, diarrhea 4% vs. 1%, increased lipase 6% vs. 1%, and asthenia 1% vs. 2%. Severe adverse events occurred in 30% of experimental arm patients and 18% of control arm patients. The percentage of toxic deaths in the two arms was similar, 2%. Efficacy and safety data support a favorable risk–benefit profile for nivolumab plus ipilimumab combined with chemotherapy as first-line treatment for patients with advanced NSCLC: median OS was 14.1 months in the experimental arm versus 10.7 months in the standard therapy arm, mPFS of 6.8 months versus 5 months, and an ORR of 37.7% versus 25.1% (2). The most important limitation of the study is the chemotherapy control arm, which does not represent the standard of care due to treatment advances during the trial. At this year’s ASCO annual meeting, the 4-year update of the Checkmate 9LA study confirmed that the overall risk of progression is reduced by 26%, with 21% of patients treated with chemoimmunotherapy combination alive compared to 16% of patients treated with chemotherapy alone at 4 years. The survival curves continue to show an advantage in favor of the chemoimmunotherapy combination in all subgroups of PD-L1 expression and isotype. The benefit appears to be more significant in the negative PD-L1 subgroup and the squamous isotype, with a 34% and 36% reduction in the risk of progression, respectively (10).

The *post-hoc* analysis reveals a persistent efficacy in patients who discontinued treatment due to treatment-related adverse events. Median OS was 27.5 months, ORR was 51%, and 56% of responders had an ongoing response 1 year after discontinuation, demonstrating a beneficial effect over time of the activation stimulus of the immune system, at different points, through the use of two drugs. In patients who stopped therapy due to adverse events related to treatment, median TFI was 10.6 months, with 27% of patients living and free from treatment 4 years after discontinuation of study therapy (11).

## Conclusions

The therapeutic value of ICIs has been found and recognized in several tumors, and although ICIs have improved the prognosis of advanced diseases, sometimes they cause irAEs. Our experience highlights the importance of prompt suspicion, diagnosis, and early intervention of these fatal irAEs after receiving immunotherapy, and the reasonable treatment of patients are very important for the prognosis of patients. Our case shows that ICI-associated myocarditis–myositis–myasthenia gravis, which occurs early during therapy and more frequently with combination regimens, represents a severe irAE with a high mortality rate. Because symptoms can be overlooked, especially in sick patients, our observations suggest the need for a high index of suspicion among oncologists. Because of the rarity of ICI-associated myocarditis–myositis–myasthenia gravis, more high-quality evidence is necessary to establish reliable guidelines for the future.

## Data Availability

The original contributions presented in the study are included in the article/supplementary material. Further inquiries can be directed to the corresponding author.
